# The transformative potential of lipid nanoparticles tailored for acute myeloid leukemia immunotherapy

**DOI:** 10.3389/fimmu.2026.1799525

**Published:** 2026-09-07

**Authors:** Alexander Kamb, John S. Welch

**Affiliations:** 1 Discovery Research, A2 Biotherapeutics, Agoura Hills, CA, United States; 2 Clinical Development, A2 Biotherapeutics, Agoura Hills, CA, United States

**Keywords:** cell therapy, LNPs, logic gate, mRNA, multiple myeloma, NHL, tumor-associated antigen, leukemia

## Abstract

T-cell engagers and engineered cell therapies have yet to deliver the same spectacular results in acute myeloid leukemia (AML) that they have in other hematologic malignancies. In this review, we analyze the challenges with AML immunotherapy development, including on-target, off-tumor toxicity, cytokine release syndrome (CRS), and the “myeloid sink.” We then discuss the emergence of two technologies that could address the major challenges with investigational AML therapeutics: targeted, lipid nanoparticles (LNPs) loaded with specific mRNAs and a type of synthetic logic gate (a NOT gate). This approach builds on recent advancements of mRNA-loaded LNPs that developed with the COVID19 vaccines and evolved into vehicles for delivery to T cells. The LNP modality offers a new opportunity for effective treatment of patients with AML.

## 1 Introduction

Acute myeloid leukemia (AML) is a common blood cancer of the elderly, with a median age at diagnosis of 68; approximately 11,000 people in the United States and over 130,000 people worldwide die of AML annually (1). AML originates from the early progenitors of the myeloid hematopoietic lineage and encompasses a variety of forms ranging from partially differentiated cells that resemble monocytes to poorly differentiated blasts (2). Outside of two molecularly defined subtypes (M3-acute promyelocytic leukemia with 15;17 translocation and perhaps NPM1/IDH co-mutant AML), stem cell transplant (SCT) remains the only modality with long-term responses consistent with cure. Only half of patients with AML in the US qualify for SCT, and roughly half of these transplant recipients ultimately relapse. The therapeutic options for those too old or otherwise unqualified for transplant are largely ineffective, with a 5-year survival of about 10%.

Such poor outcomes occur not because we lack a sophisticated understanding of the molecular basis of AML (3); rather, the dismal survival rates result from known factors that challenge drug discoverers and clinicians (4; see next section). Indeed, the challenges of investigational medicines in AML have compelled many drug discovery organizations to abandon investment in novel therapies. In contrast, oncologists specialized in other blood cancer types, notably non-Hodgkin lymphoma (NHL) and multiple myeloma (MM), choose from an increasing menu of therapeutic options, including highly effective new modalities such as T-cell engagers (TCEs) and cell therapy (5, 6; see 7 for a review of AML cell therapy).

So why is AML a particularly difficult blood cancer to treat? Translational studies over recent years exposed many of the reasons that investigational medicines have failed (**Table 1**). In the following sections, we review the causes for the failures of AML immunotherapies and describe how integration of two relatively new therapeutic technologies, synthetic logic gates and mRNA-loaded lipid nanoparticles (LNPs), provides a plausible solution to each of these obstacles. Although there is not yet direct evidence for the combination of these modalities (LNPs and logic gates) in AML, we argue that together they offer several conceptual advantages and are worth investigating.

**Table 1:** Tmod AML design rationale.

Challenges for AML immunotherapy	Causes	AML tLNP mitigations
On-target, off-tumor toxicity	Overlap between antigens on tumor and normal cells. No new antigens to find.	New ways of using known antigens. Tmod NOT gate blocker to protect HSCs, etc.
Cytokine release syndrome	Overactivation of T cells. Tumor lysis syndrome. Overactivation/stimulation by and of normal myeloid cells.	Blocker(s) to protect normal cells. Graded intra-patient dose escalation. mRNA LNPs to limit duration. CD8-targeted LNPs.
Chemotherapy and lymphodepletion toxicity	Need for tumor induction. Need for engraftment (SCT and CAR-T).	T cells engineered *in situ* with LNPs.
Limited potency	Dose-limiting toxicity limits dosing and boosters. T-cell exhaustion by high tumor burden. Low number/quality of patient T cells.	Minimization of CRS. Minimization of killing of normal cells. Re-dose LNPs. Higher quality of T cells modified *in situ*.
Antigen escape	Heterogeneity of AML. Selective pressure for resistance to therapy.	Choose antigens for maximum efficacy. Combine antigens.
Hostile tumor microenvironment	Cellular/biochemical factors that impede inflammatory process. Factors that affect tumor cell survival.	Re-dose LNPs. Add boosters that enhance cytotoxicity.

Tmod targeted LNPs address the problems that have stymied immunotherapy for AML. AML, acute myeloid leukemia; CAR-T, chimeric antigen receptor T cell; CD8, cluster of differentiation 8 (referring to CD8+ cytotoxic T cells); CRS, cytokine release syndrome; HSC(s), hematopoietic stem cell(s); LD, lymphodepletion; LNP(s), lipid nanoparticle(s); SCT, stem cell transplant; tLNP, targeted lipid nanoparticle.

## 2 The challenges of AML immunotherapy

### 2.1 On-target, off-tumor toxicity

A major obstacle of new cancer treatments is the rarity of truly tumor-specific targets for the “magic bullets” that have been sought intensely since they were envisioned by Paul Ehrlich in 1908 (8). Although a few such cancer targets have been found (e.g., BCR-ABL in chronic myeloid leukemia), these are rare and/or impractical to exploit therapeutically. Despite the notable success of immunotherapeutics in both NHL and MM, the targeted antigens are not uniquely expressed on tumor cells (e.g., CD19, CD20, CD38, and BCMA). Rather, these antigens are specific to B-cell lineages and mark both malignant and normal cells for destruction. The key to therapeutic success is not tumor-specificity of antigens, but rather the fact that patients can survive for months without B cells. Because AML cells are closely related to essential cell types, such as neutrophils and hematopoietic stem cells (HSCs), immunologic targeting of cells expressing AML antigens can result in myeloid cytopenias and even aplasia. Whereas prolonged B-cell cytopenia can be manageable, neutropenia lasting more than 4–6 weeks can lead to life-threatening fungal and bacterial infections.

Several antigens have been identified that have varying degrees of AML specificity and homogeneity of expression within AML tumors. These antigens include CD33, FLT3, CLL-1 (CLEC12A), and CD123 (IL3RA). Immunotherapeutics targeting each of these have been studied in clinical trials with mixed success (**Table 2**). The details vary from study to study, but consistent patterns of toxicity and limited efficacy have been observed, as described below.

**Table 2:** Summary of clinical trials of CAR-Ts or TCEs for AML.

Study institution)	Modality	Indication [no. patients treated]	LD	Doses received [no. patients in dose group]	Safety [no. patients with event]	Efficacy [no. patients with event]	Comments
Target: CD33							
NCT05105152 Appelbaum et al, 2024 (Fred Hutch) (9)	SC-DARIC33, fully human, rapamycin-regulated CD33-directed CAR-T	pediatric and young adult patients with R/R AML [3]	flu/cy (doses undisclosed)	SC-DARIC33: 1×10^6^ cells/kg with rapamycin 0.5 mg/m^2^ daily oral dose on Days 3–21 [3]	markers associated with CAR-T-cell activation and potential CRS	early signs of antitumor impact: chloroma necrosis [1] 99.8% reduction in circulating blast-like CD33-high cells [1]	preclinical data demonstrated the DARIC33 T cell function paused when rapamycin was withdrawn and restored with rapamycin re-exposure
NCT01864902 Wang et al, 2015 (Chinese PLA General Hospital) (10)	autologous CD33-directed CAR-T	R/R AML and long-term pancytopenia [1]	none	a total of 1.12×10^9^ cells (over 4 doses across 4 days) [1]	CRS [1] grade 4, exacerbation of pre-existing pancytopenia [1]	no responses; bone marrow blast reduction	
NCT05984199 Shah et al, 2024 and Mushtaq et al, 2026 (Vor Bio) (11, 12)	VCAR33, donor-derived CD33-directed CAR-T	adults with relapsed or MRD+ CD33+ AML who had HLA-matched allogeneic HCT [15]	flu 120 mg/m^2^ cy 1000 mg/m^2^	Arm A (bone marrow blasts ≥5%): DL1: 1×10^6^/cells/kg [7] Arm B (bone marrow blasts <5%) at DL1 [5] and DL2 (3×10^6^/cells/kg) [3]	CRS [14] (grade<3) ICANS [1] (grade≥3) GVHD [1] (grade 3)	Arm A: CRi [2] Arm B: MRD clearance [1] transient VCAR33 expansion [14]	manufactured using lymphocytes from each patient’s prior allogeneic stem cell donor
NCT03927261 Sallman et al, 2022 (Precigen) (13)	PRGN-3006 UltraCAR-T, autologous CD33-directed CAR-T	R/R AML [20] CMML [1] MDS [3]	Cohort 1: none Cohort 2: Days -5 to -3: flu 30 mg/m^2^ cy 500 mg/m^2^	Cohort 1: 1.8 to 50×10^6^ [10] Cohort 2: 4.4 to 83×10^6^ [14]	no deaths or DLTs Cohort 1: CRS: grade 1 [3] grade 2 [3] grade 3 (transient) [1] Cohort 2: CRS: grade 1 [7] grade 2 [3]	Cohort 1: no responses Cohort 2: CRi [1] CRh [1] PR [1] bone marrow blast reduction [7]	mem-IL-15 onboard; cells manufactured overnight
NCT03915379 Narayan et al, 2024 (Janssen) (14)	JNJ-67571244 (CD33×CD3 TCE)	R/R AML [56] R/R MDS [12]	none	twice‐weekly IV 0.2 to 37.5 μg/kg [56] once‐weekly SC 6.3 or 12.6 μg/kg [12]	grade≥3 AEs related to study therapy: CRS [7] ICANS [2] GGT increased [11] cardiac disorders [4] DLTs [11]	no responses	study terminated due to minimal clinical activity
Target: CLL-1							
ChiCTR2000041054 Jin et al, 2022 (Nankai University) (15)	CLL-1 CAR-T	adults with R/R AML [10]	3 days: flu 30 mg/m^2^ cy 500 mg/m^2^	1-2×10^6^/kg [10]	CRS: all grade [10] grade≥3 [6] (severe pancytopenia)	CR [1] CRi [6]	
NCT03222674 Zhang et al, 2022 (Bio-gene) (16)	autologous anti-CLL1 CAR-T	children with R/R AML [8]	3 days: flu 25–30 mg/m^2^ cy 500–900 mg/m^2^	0.35-1×10^6^/kg [8]	CRS [8] grade 1-2 pancytopenia [8] grade 3-4	CRi MRD- [4] CRi MRD+ [2] PR [1]	findings suggest patients with more than 90% of CLL1 positivity on AML blasts may benefit from CLL1-CAR-T treatment
NCT06017258 Geyer et al, 2025 (17)	CD371/SAVVY/IL-18 CAR-T	adults with R/R AML [5]	3 days: flu 30 mg/m^2^ cy 500 mg/m^2^	3×10^4^/kg [3] 3×10^5^/kg [2]	CRS: grade 1–2 [3] grade 3 [1] grade 4 [1] ICANS [1] grade 3 DLTs [2] (prolonged cytopenias, marrow hypoplasia, severe CRS)	MLFS MRD- [3]	findings suggest IL-18 secreted from proliferating CAR-T cells promoted cytotoxic T and NK cell expansion
Target: CLL-1/CD33							
NCT03795779 Liu et al, 2018 (iCell Gene, iCAR Bio) (18)	cCAR, CLL1-CD33 compound CAR-T	pediatric [1] and adult [8] R/R AML	flu 30 mg/m^2^ cy 300 mg/m^2^	1-3×10^6^/kg [9]	CRS: grade 1–2 [6] grade 3 [2] ICANS [4] grade 3 pancytopenia [8] grade 4	MRD- [7]	
Target: CD123							
NCT03766126 Bhagwat et al, 2024 (Penn, Novartis) (19)	CART-123, autologous anti-CD123 CAR-T	adults with R/R AML [12]	flu/cy (doses undisclosed)	5×10^5^/kg [1] 8×10^5^/kg [1] 2×10^6^/kg [10]	CRS [10] DLT and death from CAR-T-related toxicity [2] (probable grade 5 CRS)	CR [2] CRi [1]	inhibition of JAK/STAT signaling restored CAR-T sensitivity
NCT02159495 Budde et al, 2020 (Mustang Bio) (20)	CD123CART/MB-102, donor-derived or autologous anti-CD123 CAR-T	R/R AML [7] BPDCN [2]	3 days: flu 25−30 mg/m^2^ cy 300−500 mg/m^2^	5×10^7^ to 2×10^9^ [9]	no grade≥3 CRS, neurotoxicity, or DLTs	AML: CRi [1] MLFS [2] BPDCN: CR [1]	
NCT04230265 Wermke et al, 2023 (aviCell UniCAR) (21)	AVC-101 (UniCAR02-T-CD123), CAR-T adaptor	R/R AML or MRD+ AML [19]	3 days: flu/cy (dose undisclosed)	multiple cycles of UniCAR-T 5×10^8^ [19]	CRS: grade 1 or 2 [9] grade 3 [3] ICANS [1] (grade 2) DLT (reversible drop in fibrinogen levels) [1]	OR: R/R AML [8] MRD+ AML [3]	still dose-escalating
NCT02730312 Ravandi et al, 2023 (Xencor) (22)	Vibecotamab, CD123×CD3 TCE	adults with: AML [118] ALL [1] CML [1]	none	varying schedules of priming, step-up, and target doses ranging from 0.003 μg/kg to 12.0 μg/kg [111] RP2D: 500 ng/kg/day	CRS: grade 3 [27] grade 4 [4] grade 5 [1] (related to study treatment) DLTs: CRS [2] IRR [2] GGT increase [2] hypertension [2]	AML: CR [3] CRi [3] MLFS [3] PR [1]	responders to the treatment were associated with statistically significant lower absolute blast counts (<25%) at baseline in both the blood and bone marrow
NCT02152956 Uy et al, 2021 (MacroGenics) (23)	Flotetuzumab, CD123×CD3 TCE (DART)	adults with: R/R AML [50] PIF/ER AML [30]	none	RP2D: 500 ng/kg [88]	grade≥3: CRS/IRR [8] platelet count decreased [14] lymphocyte count decreased [9] DLTs [3] (grade 3 delirium, grade 3 acute confusion, grade 3 CRS)	R/R AML: CR [6] CRh [3] CRi [1] MLFS [2] PIF/ER AML: CR [5] CRh [3] CRi [1] MLFS [1]	increased response in patients with inflamed TME 10-gene immune signature strongly predicted a complete response to the therapy
Target: CD33/FLT3							
NCT06325748 Farhadfar et al, 2025 and Muftuoglu et al, 2025 (Senti Bio) (24, 25)	SENTI-202, logic-gated CAR NK cell therapy targeting CD33/FLT3	adults with R/R AML [12]	5 days: flu 30 mg/m^2^ cytarabine (Ara-C) 2 g/m^2^	1×10^9^ [6] 1.5×10^9^ [6]	CRS [2] grade 1-2 grade≥3 AEs related to study therapy: febrile neutropenia and platelet count decreased [1] no DLTs	CR MRD- [4] CRh MRD+ [1] MLFS MRD+ [1] in responders, >10-fold decrease in leukemic stem cells	designed with an inhibitory NOT gate CAR for endomucin to selectively spare healthy hematopoietic stem/progenitor cells

AACR, American Association for Cancer Research; AML, acute myeloid leukemia; BPDCN, blastic plasmacytoid dendritic cell neoplasm; CAR-T, chimeric antigen receptor T cell; CLL-1, C-type lectin-like molecule-1; CMML, chronic myelomonocytic leukemia; CR, complete response or complete remission; CRh, complete remission with partial hematologic recovery; CRi, complete remission with incomplete hematologic recovery; CRS, cytokine release syndrome; DLT, dose-limiting toxicity; FLT3(L), FMS-like tyrosine kinase 3(ligand); flu/cy, fludarabine and cyclophosphamide; GGT, gamma-glutamyl transferase; GVHD, graft-versus-host disease; HSC(s), hematopoietic stem cell(s); HCT, hematopoietic cell transplantation; ICANS, immune effector cell-associated neurotoxicity syndrome; IRR, infusion-related reactions; IV, intravenous; LD, lymphodepletion; mem-IL-15, membrane-bound interleukin 15; MDS, myelodysplastic syndrome; MLFS, morphologic leukemia-free state; MRD (–)/MRD(+), measurable (or minimal) residual disease negative/positive; NK, natural killer; OR, objective response; PIF/ER, primary induction failure/early relapse; PR, partial response; R/R, relapsed/refractory; RP2D, recommended phase 2 dose; SC, subcutaneous; SCT, stem cell transplant; TCE, T-cell engager; TME, tumor microenvironment.

Clinical data from clinical trials of CAR-Ts or TCEs for AML suggest all targets have toxicity risk. It is difficult in most cases to disentangle the effects of preconditioning lymphodepletion from the cell therapy product. [For a systematic review of CAR-T therapy for AML, see (7)].

### 2.2 Cytokine release syndrome

A common type of toxicity observed with the more potent types of immunotherapeutics (i.e., TCEs and chimeric antigen receptor T cells [CAR-Ts]) is CRS, a form of systemic inflammation characterized by immune cell activation, proliferation, and high levels of blood cytokines (26). Although not exclusive to AML therapy, CRS has been especially problematic in AML and often hampers dosing, or worse, can manifest with such severity that it causes trial termination and even patient deaths (27). Although the mechanism of CRS is not fully understood, certain aspects are clear:

1. CRS only occurs in the presence of antigen and is therefore considered a form of on-target toxicity that does not directly cause destruction of healthy tissues (28); rather, it can induce a wind-up of immune activation with non-specific neurologic symptoms called immune effector cell-associated neurotoxicity syndrome (ICANS) or engulfment by macrophages in the liver, spleen, bone marrow, and lymph nodes, described initially as hemophagocytic lymphohistiocytosis (HLH) and more recently as immune effector cell-associated hemophagocytic lymphohistiocytosis-like syndrome (IEC-HS) (29). Both of these extreme forms of CRS are rare but can be a fatal consequence of CAR-T treatment.
2. All antigens tested in AML so far (CD33, FLT3, CLL-1, and CD123) seem to induce CRS when targeted by T cells in at least some trials, suggesting it is not specific to one antigen type (see **Table 2**).
3. CRS responds to glucocorticoids and IL-6 antagonist antibodies (26), but these immune-system-dampening remedies may impact immunotherapy efficacy.
4. At least for immunotherapy of B-cell malignancies, CRS generally is dose-dependent and more pronounced in patients with heavy tumor burden (30).
5. CRS has been observed in clinical studies and toxicology studies in primates at high doses of CAR-Ts where tumors are not present (31–33). Thus, expression of antigen on healthy tissues is sufficient to trigger CRS.
6. With TCEs like blinatumomab, mosunetuzumab-axgb, and flotetuzumab, CRS can be reduced by gradual ramping up the infusion dose, suggesting the kinetics of exposure are important (26). The T cells that initiate the process may desensitize and/or traffic out of the blood where they are less likely to encounter additional TCE molecules.
7. The magnitude and manifestation of CRS likely depend on the tumor type targeted by the T cells (26).

In AML, the origin of the hyper-inflammatory cycle is not established. Although it likely begins with antigen-dependent activation of T cells, it is not certain if inflammation escalates because of:

1. The scale of direct T-cell activation by the antigen.
2. The release of cytokine granules from the targeted tumor cells that can be numerous in some patients; and/or
3. The on-target, off-tumor killing of normal myeloid cells.

Although cell therapies using non-T-cell effectors (e.g., natural killer [NK] cells) target the same antigens, they induce lower cytokine levels, suggesting that T-cell overactivation primarily drives this escalating inflammation (24). CRS may be a property of T cells that correlates with their potency as cytotoxic effectors.

### 2.3 Complications from chemotherapy and lymphodepletion

AML is treated by induction with chemotherapeutics to reduce tumor burden and, in the case of SCT, to eliminate the host immune system in preparation for engraftment. Although necessary—at least for SCT—LD is a substantial toxicity burden on patients that increases risk of infection. In addition, common LD chemotherapies (e.g., fludarabine and cyclophosphamide) are active in AML, creating a confounding variable in the interpretation of clinical results that clouds conclusions about the role of the investigational therapeutic in efficacy and toxicity. For example, after treatment with CAR-Ts, patients with AML often display marrow aplasia/hypocellularity. Is this the result of LD, on-target elimination of bone marrow cells, or HLH? Does exposure to LD before CAR-T induce additional susceptibility to HSCs and risk of aplasia, and can this be mitigated (17)? The fact that TCEs targeting the same antigens do not produce identical levels of hypocellularity may suggest that some of the depletion observed with CAR-Ts is not on-target (34). Caveats of this interpretation are: (i) the low blood concentrations that TCE agents can achieve due to their typical dose-limiting toxicity (DLT); and (ii) the absence of LD in TCE regimens.

LD increases the risk of infection both by lowering blood cell counts and by causing tissue damage, particularly altering gut integrity and permitting bacterial translocation. Patients with AML often have marginal neutrophil counts, and chemotherapy decreases their ability to tolerate infections and increases the risk of translocating bacteria through gut toxicity. When comparing patients with AML or myelodysplastic syndrome (MDS) treated with supportive care vs. hypomethylating agents, the administration of chemotherapy is associated with greater infection risk, suggesting that non-hematopoietic tissue damage caused by the chemotherapy may augment infection risk beyond the risk observed from tumor-associated cytopenias (35, 36). Likewise, infections have not been reported as a recurrent complication of TCEs in AML, which have been given in the absence of chemotherapy (14, 23), suggesting that strategies avoiding chemotherapy may avoid infection risk, even if they worsen cytopenias.

### 2.4 Lack of potency

Potency is the dose-related therapeutic efficacy. Multiple cell- and TCE-regimens have demonstrated activity, but responses have not been durable and generally have not resulted in sufficient immunologic pressure to induce recurrent antigen loss at relapse (37). Potency of immunotherapeutics is a combination of at least two factors: (i) sensitivity to activation by antigens, and (ii) proliferation which increases the effector:target cell ratio. Sensitivity typically manifests as acute activity, cytokine production, cytotoxicity, and short-term proliferation. Potency can also be a function of overall exposure to the therapeutic, a result of survival, proliferation, and persistent activity of effectors (e.g., T cells). Indeed, the enduring success of adoptive cell therapies relies on the persistence of memory T cells, which possess robust self-renewal and proliferation capabilities (38). One of the key variables to track in immunotherapeutics is exhaustion—the decline of functional capability of effector cells after the initial phase of activation (discussed below).

Immunotherapeutic modalities are capable of extreme potency. For example, TCEs can achieve femtomolar activity *in vitro* (39). T cells engineered to express CAR- or T-cell receptor (TCR) can attain sensitivities that are almost incomprehensible, able to kill cells that carry on average only a few—possibly as low as one—antigen molecule (40).

Low potency can be compensated for by administering higher doses. However, two factors limit the doses that can be studied, one technical and one biological. On the technical side, it is sometimes unfeasible to produce or administer sufficiently large numbers of cells or proteins for purely practical reasons. In humans, the limit of administration is around 1×10^11^ cells, a huge number exceeding by 10-fold the normal number of T cells that circulate in the blood, especially considering that a normal adaptive immune response starts from a single cell. For proteins, the maximum feasible dose is typically about 1 gram, but most TCEs are so potent and toxic, that this level is far below the maximum tolerated dose. The second factor is DLTs caused either by CRS or on-target, off-tumor toxicity. This issue, confounded by the effects of LD, can confuse estimation of true potency. Doses of CAR-Ts (and TCEs) in patients with AML are in the range of those used for other indications and well below the level given for tumor-infiltrating lymphocytes, which can exceed 1×10^11^.

Exhaustion, a major source of potency loss in the long term, is an important feature of immunotherapeutics in general and of AML therapy in particular. Exhaustion occurs during natural T-cell responses to antigen, one of the many mechanisms that guard against overactivation and autoreactivity. It is thought to underlie much of the anergy that blunts the immune response to neoantigens in cancer and is the favored mechanism by which checkpoint inhibitors work (41). In AML immunotherapy, CRS-induced cytokines have been suggested to augment exhaustion phenotypes while supporting tumor survival, compounding the problem (19). Functional characterization of CAR-Ts from patients post infusion poses challenges but has been studied in a few translational experiments with solid tumor cell therapy. For example, engineered cells in three patients infused with a CEA TCR-T were detected at levels **>**5% of circulating T cells for many days, but functional reactivity to antigen diminished much more rapidly (42). Patients with AML typically have fewer T cells due to their age, the nature of their disease, and/or previous chemotherapy. Thus, there may be a disproportionate therapeutic benefit for approaches that harness high-quality cell subsets in autologous settings to maximize the available effectors.

The so-called “myeloid sink” has also been proposed as a barrier to immunotherapy success in AML. Because AML-associated antigens are also expressed on HSCs, myeloid progenitors, and monocytes, these healthy cells act as a “sink” that diverts the therapy away from the tumor. This increases the overall target burden and tilts the effector:target ratio against the immunotherapeutic. Furthermore, these cells appear more resistant to CAR-mediated killing (43), and increased cell cycling of HSCs and myeloid progenitors following LD may further increase cell numbers (44).

### 2.5 Antigen escape

AML is possibly the most heterogeneous of all neoplasms. Even before the advent of molecular analysis, histopathology recognized multiple subtypes based on morphological features and cytogenetics. Now, with the application of DNA sequencing, at least eight categories are routinely used to classify subtypes (45, 46). This heterogeneity creates major questions for AML therapy: (i) can a single therapeutic be sufficient to treat all or most patients with AML? and (ii) will intra-patient tumor heterogeneity cause relapse of antigen-negative tumor subclones? Given the pre-existing heterogeneity of AML and the documented evolution of resistant clones in patients treated with small molecule therapeutics, *a priori* one might expect AML to escape readily from immunotherapy via antigen loss, as has been observed in other hematopoietic malignancies when immunologic pressure is applied to a single antigen (47). However, this has not yet emerged as a recurrent phenotype at relapse. For example, in a recent study of five patients using a CD123 CAR-T, relapse occurred in all three responding patients (16). Yet all of these emergent clones retained antigen, suggesting that some other factor was involved, possibly CAR-T exhaustion. A dual-targeting CLL1-CD33 CAR-T study reported consistent responses, potentially overcoming the risk of antigen escape, but the therapy was limited by high-grade pancytopenia and CRS (18).

### 2.6 Hostile tumor microenvironment

The local environment in which tumor cells grow in the body has long been considered a potential source of treatment resistance. In solid tumors, one of the differences noted early on by Otto Warburg was the hypoxic, acidic nature of the solid tumor tissue relative to healthy tissues (48). Cells that comprise the normal components of tumors (stroma), especially tumor-associated fibroblasts and macrophages, have been proposed to contribute to a hostile TME of solid tumors and to interfere with immunotherapeutics. AML re-organizes the bone marrow cytokine milieu toward myeloid cytokines. Compared with bone marrow from healthy volunteers, AML has been reported to have increased levels of proinflammatory and myeloid-supportive cytokines, including TGF-β (reviewed in 49–51). This myeloid-shift of the microenvironment supports malignant myeloid cell survival and may be detrimental to lymphoid effectors targeting AML. Furthermore, therapies that induce strong CRS generate cytokines like GM-CSF, which can simultaneously impair CAR activity and promote AML cell survival (19).

Although CAR-T cells traffic into the bone marrow and other AML sites, and efficient trafficking correlates with activity (52), the process of manufacturing can negatively impact trafficking and access to AML cells *in vivo*. Specifically, during CAR-T manufacturing, *ex vivo* cell growth conditions result in reduced expression of CXCR4, a critical chemokine receptor necessary for bone marrow cell trafficking (53). Conversely, overexpression of CXCR4 has been demonstrated to improve *ex vivo* manufactured CAR-T bone marrow trafficking and could improve activity (54).

## 3 Solutions provided by targeted mRNA LNPs tailored for AML

### 3.1 The emergence of targeted LNPs as a new modality

**Figure 1:**
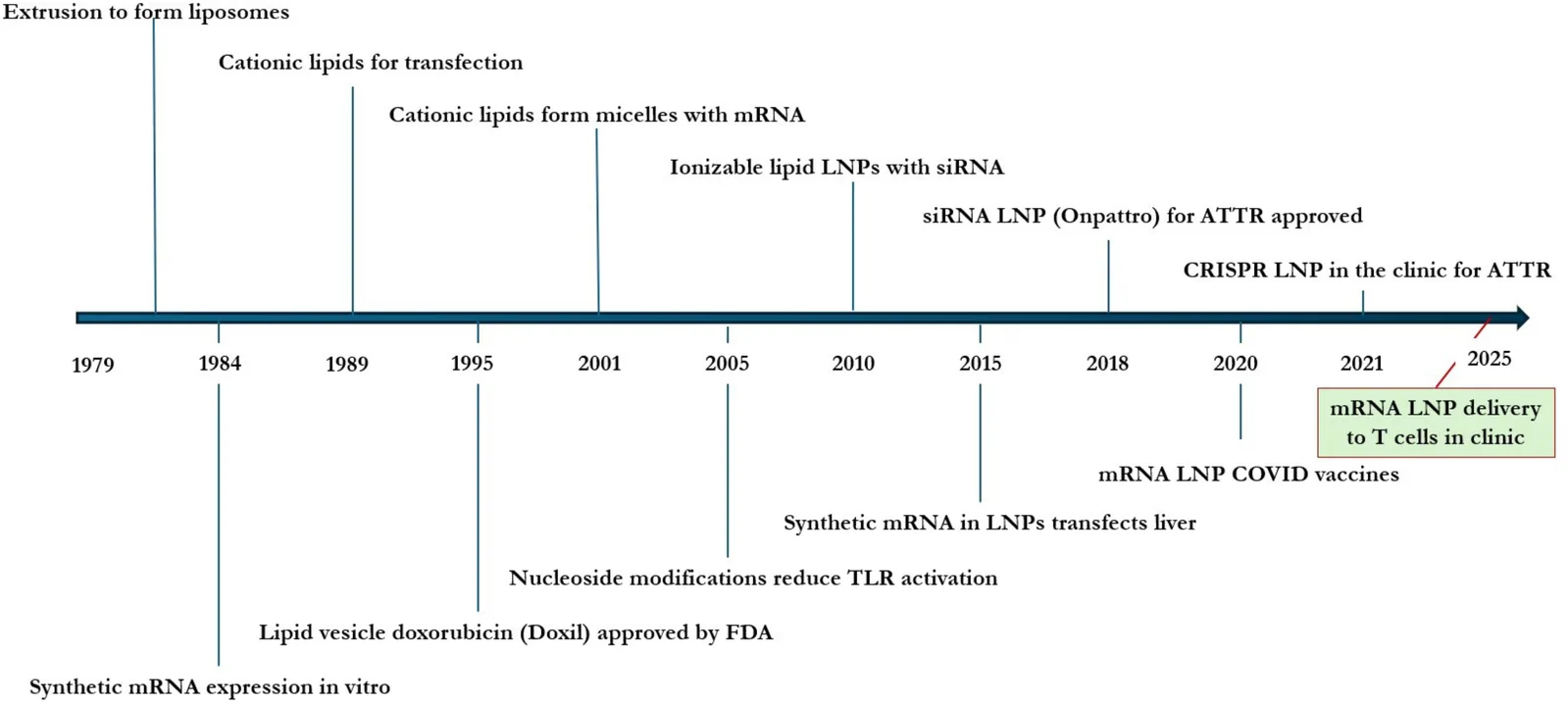
Milestones in the development of LNPs that target T cells.

Nucleic acid LNPs have come a long way since they were first introduced as reagents for DNA transfection in 1989 (**Figure 1**; 55). Two key innovations underlie the success of mRNA-loaded targeted LNPs (tLNPs) as T-cell engineering agents during the past year (32; for review see 56). The first innovation is the incorporation of ionizable lipid into the LNP in 2010 (57). These lipids are designed for two features: (i) they complex with the phosphates on nucleic acid payloads when protonated; and (ii) because their pKa < 7, the uncomplexed basic group protonates in endosomes and interacts with phospholipid head groups on the vesicle, ultimately enabling fusion and cargo-unloading into the cytoplasm.

The second innovation involves the incorporation of polyethylene glycol (PEG) polymers that serve two purposes: (i) creation of an inert layer that physically interferes with concentration of LNPs in the liver; and (ii) provision of convenient attachment sites for targeting ligands; e.g., antibodies. As long as certain stoichiometries are retained in the formulation, the combination of nucleic acid, cholesterol, phospholipid, ionizable lipid, and PEG creates a robust platform for delivery of genetic payloads to immune cells. Such tLNPs, if conjugated with T-cell surface antigen binders, efficiently modify T cells *in situ* in mice, macaque monkeys, and humans (32, 58).

T-cell directed tLNPs overcome major obstacles that hampered earlier LNPs. They reduce liver bias and toxicity and improve the efficiency of uptake and cargo-unloading by T cells. Production at scale of tLNPs is now feasible and certainly far cheaper than most engineered cell therapy products (e.g. CAR-T). tLNPs have the potential to improve the quality and potency of the T-cell response due to the utilization of T cells *in situ* and the avoidance of cell-stressing procedures for production *ex vivo*. tLNPs can be directed to subsets of cells (e.g., T cells vs NK cells, CD4 vs CD8 cells, etc.) to provide restricted types of immunologic activity. Lastly, tLNPs using mRNA transcripts, absent integration, result in transient *in vivo* CAR emergence. As discussed below, these and other features of tLNPs offer opportunity in the context of AML. But first, we describe the value of an mRNA payload that encodes a logic gate, specifically the Tmod NOT gate, for an AML tLNP.

### 3.2 The integration of a synthetic NOT gate with tLNPs

**On-target, off-tumor toxicity**: The problematic nature of tumor-associated antigens that is the root cause of on-target, off-tumor toxicity in AML will not be resolved, in our opinion, through discovery of new, improved AML antigens, given the enormous efforts over the past decades to scour the genome. Indeed, the best, most AML-specific antigens have been known for years: CD33, linked to AML in 1984 and the target of the first ADC (gemtuzumab ozogamicin; 59); FLT3, associated with AML in 1996 (60); CD123, discovered as an AML antigen in 2000 (61); and CLL-1, identified in 2004 (62). After decades of searching for better antigens, we must invent novel ways to exploit these well-known targets.

**Figure 2:**
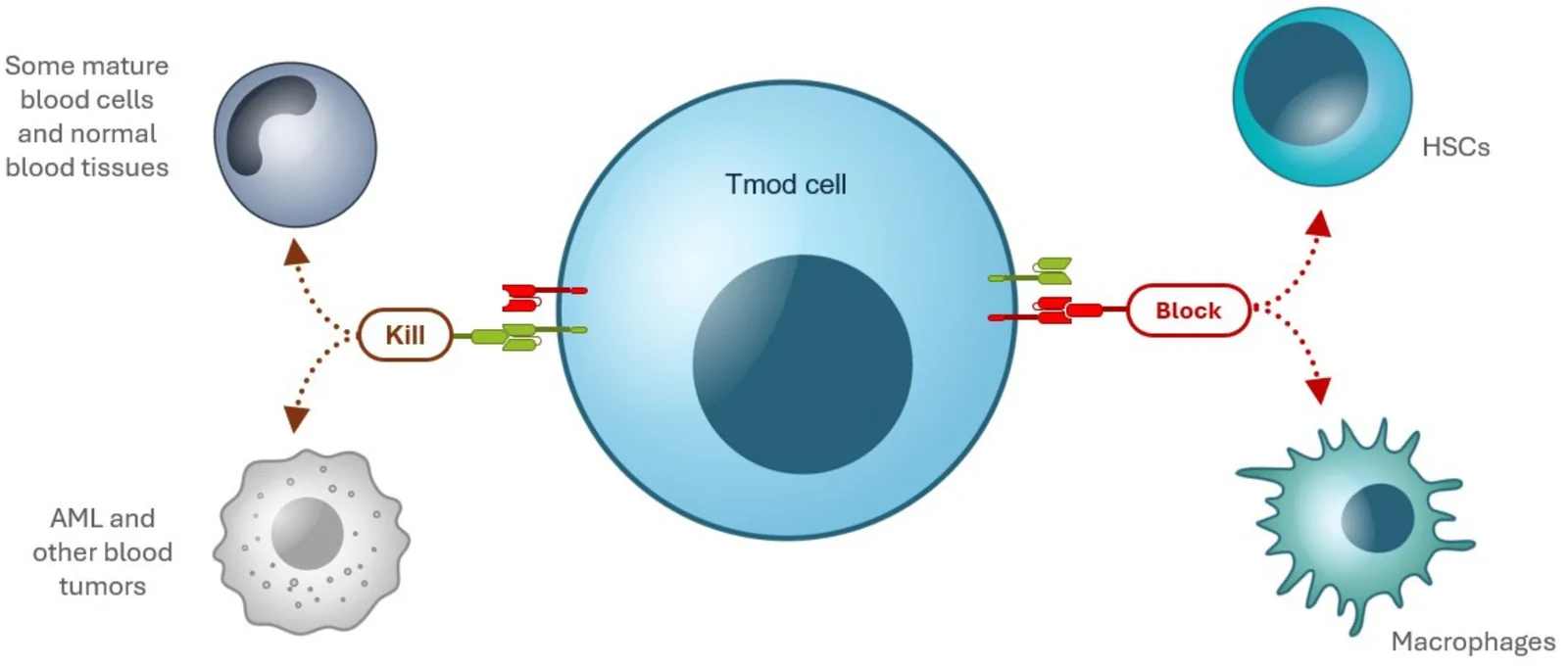
Tmod dual-receptor NOT gate solution for AML and other blood cancers. The activator (blue) activates T cells in the presence of a target A-antigen, but is overridden by the blocker (red) when both the activator antigen(s) and blocker antigen are present, allowing selectivity of targeted cell types (tumor) and protection of normal cell lineages (e.g., HSCs). In this case, the activator is a tandem construct with two scFvs, each one recognizing a different activator antigen.

In principle, tumor selectivity may be generated by a synthetic circuit that recognizes antigen profiles, rather than single antigens, to differentiate pathologic cells from other closely related cell types (63). An especially useful circuit involves a dual-receptor NOT gate called Tmod that can be designed to protect essential healthy cell types that otherwise would be eliminated by simple CAR-Ts targeting pathologic cells (64) (**Figure 2**). The Tmod system can differentiate among cell types based on both the presence and absence of specific antigens, expanding T-cell antigen-response beyond single-antigen-driven clonal selection. The mechanism of discrimination relies on two receptors: (i) a CAR (activator) that triggers cytotoxicity when specific activator antigens are present on a target cell; and (ii) an inhibitory receptor (blocker) that overrides the activator when specific blocker antigens are present. The system is modular and extensible. It can be designed to respond to multiple activator and blocker antigens (65). So far, clinical studies have confirmed that the Tmod system behaves per design, using an HLA-A*02-targeted blocker to protect healthy tissues in heterozygous patients whose tumors have lost the allele via loss of heterozygosity (LOH) (e.g., 66).

In situations like AML, it is highly advantageous to exploit more complex antigen profiles where the presence and absence of specific antigens control activation, allowing more precise discrimination among different cell types (**Figure 2**). We created a Tmod construct with a dual-CAR directed at the CD33 and CLL-1 target antigens that offers identifiable advantages as described below. However, to achieve selective depletion of pathologic AML cells and avoid HSCs, the Tmod construct could also encode a blocker directed at an antigen like CD90 to protect healthy cells that not only express the activating antigens but also express CD90 (THY1), a marker for the earliest stage of adult hematopoiesis that is rapidly lost during cellular maturation (67–69). The blocker optionally contains a second single-chain variable fragment (scFv) in tandem directed at CD16b (FCGR3B) to protect important blood lineages, including neutrophils and monocytes. Portions of this construct are described in DiAndreth et al. (64). Hypothetically, a Tmod construct with the composition CD33-CLL-1 | CD90-[CD16b] could extend the striking therapeutic success of engineered cell therapy beyond NHL and MM to AML (70). NOT gated constructs that target other antigens are also reasonable (for example see 71).

The combined benefits of the two technologies, tLNPs and Tmod, address issues beyond on-target toxicity that have impacted success of AML immunotherapy, as discussed below (**Table 1**).

**CRS**: CRS has been dose-limiting in both CAR-T and TCE modalities. tLNP CARs that integrate an inhibitory receptor offer multiple strategies to mitigate CRS for AML therapeutics. First, CRS has been associated with higher CD4 cell counts and greater CD4 expansion (72–75). tLNPs may be directed at T cells using a CD3- or CD7-targeting moiety and also offer the opportunity to bias CAR-T cells toward CD8 cells by using CD8-targeting agents. CD8-targeting may mitigate one mechanism of CRS, the overactivation of CD4 cells, although CD8 cells also contribute to CRS. Second, incorporation of an inhibitory receptor in Tmod provides cells with both activation and inhibitory signaling, breaking the cycle of continuous activation that causes T-cell overactivation and CRS. Third, like TCEs, the short half-life of tLNP-derived CARs due to the ephemeral nature of mRNA and dilution in dividing cells, offers an opportunity for pulsatile and escalated dosing, allowing T cells an opportunity to recover and reset. In this way, tLNP CARs, especially when integrated with Tmod, could boost anti-tumor activity while avoiding T-cell proliferation and the unopposed activation that drive CRS.

**Chemotherapy and LD**: For conventional *ex vivo* cell therapy, LD is thought to be a requirement to improve the engraftment of the infused engineered T cells (76). The transient nature and re-dosing potential of mRNA LNPs and tLNPs renders LD unnecessary. Studies with LNPs in monkeys and humans have shown that CD19 and 20 CARs generated *in situ* via LNPs are extremely active without LD (32, 58).

Not only is LD a major complication for the treatment of AML, it is also a confounding factor in the interpretation of clinical outcomes. Indeed, in many cases, the involvement of LD and SCT in the AML treatment protocol practically obscures the effect of the CAR-Ts. With Tmod LNPs, the acute effects of the T cells produced *in situ* will be immediately clear, and any adjustments to the dosing regimen can be implemented as quickly as investigators wish to act, within the confines of the regulations.

The Tmod tLNP strategy could be deployed in patients either pre- or post-SCT. By selectively sparing normal HSCs, the Tmod tLNP construct is designed to avoid SCT, enabling hematopoietic count recovery after dissipation of the tLNP. In this way, the transient nature of the tLNP offers an opportunity when paired with Tmod. In the context of relapse post-SCT, the transient activity of a tLNP limits long-term toxicity while allowing hematopoietic recovery, antigen spread, and an augmented graft-versus-leukemia effect from donor T cells.

**Potency**: If the Tmod LNP construct works as expected, with reduced on-target toxicity and CRS, it is likely that higher peak levels of engineered effectors can be achieved compared to previous studies of AML CAR-Ts, nearly all of which encounter DLTs. AML tumor antigens are generally expressed at levels well above the sensitivity limits of CAR-Ts, so it is probable that the high generation rate of Tmod cells *in situ* (>50% of blood T cells) and the ability to easily re-dose will result in a large total number of Tmod cells in the body (>1×10^9^). In addition, these cells may be fundamentally more active and resistant to exhaustion, compared to *ex vivo* engineered cells that have been processed in culture—grown, exposed to high levels of cytokine and other stimulants, and freeze/thawed. Finally, the potential protection of normal cells may also slow down the development of an exhausted phenotype that occurs in many *ex vivo* CAR-T and TCR-T trials (42). The approach depends on acute peak activity, not long-term responsiveness.

**Antigen escape**: The CD33 arm of the AML Tmod construct described here is chosen primarily for its genetic stability in AML samples and cell lines (65). However, to further mitigate the possibility of antigen escape, an scFv targeting a second activator antigen (CLL-1) may be added. This approach, to combine therapeutics directed at different targets, is a well-known mitigation of relapse in both infectious disease and oncology (77, 78).

Though antigen escape has so far not been consistently observed in AML studies with immunotherapeutics—likely because the responses driven by the products are not deep enough—it is possible that a potent Tmod LNP, whose dose is less constrained by the traditional sources of toxicity, will eventually drive selection of antigen-negative variants. Antigen loss is one of the major causes of relapse in other CAR-T therapies, including CD19 and BCMA CAR-Ts (79). In addition, AML is notorious for evolving escape mechanisms in response to therapy (45). Thus, to prepare for the eventuality of antigen escape and for intra-tumor heterogeneity, it may be advisable to create an AML Tmod construct that includes a tandem activator (an OR gate) targeting two antigens.

**Hostile TME**: To confront a potentially inaccessible and non-receptive TME in which AML cells thrive and tumor-directed immune cells struggle, Tmod tLNPs offer several potential benefits. The simplest advantage is the potential to re-dose in a carefully monitored fashion to maximize efficacy without undue risk to the patient. tLNPs are suited to serial dosing and in an investigational setting, the clinical and translational data should provide rapid, detailed feedback to optimize the interval. Patients with AML, either because of their disease burden or previous LD treatments, often have lower numbers of T cells, potentially also of poorer quality. However, because of the efficiency of *in situ* mRNA delivery by LNPs, it is probable these T cells will perform better than they would if they were removed and engineered *ex vivo*. Though early, the data emerging from studies in monkeys and humans support this concept (31, 32, 58).

In the event that responses are not sufficiently deep and durable, additional modules can be readily added to the basic framework of the tLNP; e.g., potency enhancers to boost sensitivity [e.g., CD40, SLP-76 domains), survival (e.g., FAS siRNA), extravasation (e.g., IL-10R or TGF-β antagonists or signal inverters; reviewed in (80)], and others.

## 4 Risks and limitations of a Tmod tLNP approach

Although the design and testing of AML Tmod tLNPs address in principle the obstacles that have impaired clinical success with AML immunotherapy, certain possible shortcomings and unknowns can be identified. With regard to the antigens, CD33 and possibly CLL-1 are expressed on HSCs; the Tmod CD90 blocker, based on abundant preclinical and clinical experience, should protect a significant fraction of HSCs. However, CD90 is not expressed on progenitor cells that are multipotent progenitor cells (MPPs), whereas CD33 is. Some of these MPPs likely will be destroyed along with the tumor. Neutrophils on the other hand live only 6–12 hours. Because CD33 and CLL-1 are expressed on some neutrophils, monocytes, and their respective progenitors, it is possible that Tmod tLNPs will deplete these cell types significantly over the course of 2 weeks, before the HSCs can regenerate them. Inclusion of an CD16b (FCGR3B) blocker should reduce this risk, but potentially at the cost of protecting AML variants that overexpress CD16b (FCGR3B). An immunotherapy product with weeks-long duration (such as an effective CAR-T) will result in prolonged cytopenias that last until the product dissipates and normal progenitors can give rise to blood count recovery. In this way, the short durability of LNP products provides an advantage, enabling transient exposure and opportunity for cyclical blood count recovery.

The transient expression of mRNA delivered via tLNPs is both a strength and potential liability. Though transient expression is a built-in safety feature, compared to genome-edited effector cells for example, the levels of protein that can be generated are limited in part by the time the mRNA persists, as well as its dilution as T cells divide post activation. The ability to re-dose offsets some of this limitation and redosing may permit an added benefit through intermittent activation and resting of the modified T cells (81).

LNPs are limited in capacity by the mass of the mRNA payload that can be encapsulated; for example, a 2 kb mRNA averages ~2.8 molecules/LNP using a standard formulation (82). Though the limits are not absolute and depend on formulation, this property will constrain the complexity of certain designs. However, depending on the design, mixtures of LNPs that carry different payloads are also possible (83).

Repeat dosing of targeted LNPs will eventually elicit an adaptive immune response that neutralizes the CD8-targeting ligand, the modified T cells generated by the tLNPs, or the PEG (84, 85). Immunogenicity is not only a theoretical risk of allogeneic cell therapies; it has been observed to coincide with the disappearance of engineered CD19 allogeneic CAR-Ts roughly 4 weeks post administration (86). With autologous immune cells, the role of host response is less clear, but certainly a risk. At the extremes, immunologic neutralization of Tmod tLNPs should not occur within the first 2 weeks, the time it takes for an adaptive response to mature. Alternatively, various immunosuppressive countermeasures, including B-cell depletion, are possible but these may increase the risk to patients. Others, such as development of nonimmunogenic targeting ligands and/or PEG substitutes may enable serial exposure of Tmod LNP without immunologic neutralization.

## 5 Relevant alternative approaches in development for AML

Other approaches to AML are under development, some of which offer certain advantages compared to previous immunotherapies. One approach also involves generation of CAR-T cells *in vivo* but employs gene-editing (e.g., *in vivo* delivery with pseudo-typed lentivirus) (87). Such a method may work in AML but it is more cumbersome and limited with regard to design. It is more immunogenic, less amenable to re-dosing, and results in genome modifications *in vivo* that create safety risks vis-à-vis T-cell transformation. A second approach utilizes allogeneic SCT to exploit mismatches at the minor histocompatibility antigen locus HA-1/2. This approach offers a clever means to take advantage of specific genetic differences between the host and graft using HA-1 or -2-targeting TCRs engineered into T cells (88). In principle, these TCRs could be delivered *in vivo* to the selected recipient. A CD33-FLT3 | EMCN engineered NK cell therapy is in Phase 1, but the use of LD and SCT obscure the activity of the cell product (25, 71). Finally, new CAR-Ts, TCEs, ADCs, and small molecules (notably menin inhibitors) continue to attract investment (89). If tLNPs demonstrate single-agent efficacy in AML, they will likely be evaluated in combination regimens, as is standard in oncology.

## 6 Conclusion

Although a variety of immunotherapies (including CAR-Ts and TCEs) have demonstrated clinical activity in AML, their overall response rates, depth, and durability remain limited. These limitations are primarily driven by severe CRS and target antigen overlap with normal HSCs and progenitor cells, which leads to prolonged cytopenias and aplasia. Through this clinical work, progress has been made toward understanding the specific causes of immunotherapy failures in AML. The Tmod tLNP construct described here provides a conceptual framework to address these persistent challenges, potentially reducing toxicity and costs while improving efficacy and convenience for patients with AML. If successful, a NOT-gated construct might be generalized to other cancers (65).
